# Memory Enhancers for Alzheimer’s Dementia: Focus on cGMP

**DOI:** 10.3390/ph14010061

**Published:** 2021-01-13

**Authors:** Ernesto Fedele, Roberta Ricciarelli

**Affiliations:** 1 Department of Pharmacy, Section of Pharmacology and Toxicology, University of Genoa, 16148 Genova, Italy; 2 IRCCS Ospedale Policlinico San Martino, 16132 Genova, Italy; 3 Department of Experimental Medicine, Section of General Pathology, University of Genoa, 16132 Genova, Italy

**Keywords:** cyclic guanosine-3′,5′-monophosphate, cGMP, phosphodiesterase, guanylyl cyclase, dementia, Alzheimer’s disease

## Abstract

Cyclic guanosine-3′,5′-monophosphate, better known as cyclic-GMP or cGMP, is a classical second messenger involved in a variety of intracellular pathways ultimately controlling different physiological functions. The family of guanylyl cyclases that includes soluble and particulate enzymes, each of which comprises several isoforms with different mechanisms of activation, synthesizes cGMP. cGMP signaling is mainly executed by the activation of protein kinase G and cyclic nucleotide gated channels, whereas it is terminated by its hydrolysis to GMP operated by both specific and dual-substrate phosphodiesterases. In the central nervous system, cGMP has attracted the attention of neuroscientists especially for its key role in the synaptic plasticity phenomenon of long-term potentiation that is instrumental to memory formation and consolidation, thus setting off a “gold rush” for new drugs that could be effective for the treatment of cognitive deficits. In this article, we summarize the state of the art on the neurochemistry of the cGMP system and then review the pre-clinical and clinical evidence on the use of cGMP enhancers in Alzheimer’s disease (AD) therapy. Although preclinical data demonstrates the beneficial effects of cGMP on cognitive deficits in AD animal models, the results of the clinical studies carried out to date are not conclusive. More trials with a dose-finding design on selected AD patient’s cohorts, possibly investigating also combination therapies, are still needed to evaluate the clinical potential of cGMP enhancers.

## 1. The cGMP Universe: Synthesis, Signaling, and Metabolism

Cyclic guanosine-3′,5′-monophosphate (cGMP) was first identified in the early ‘60s [1], but it took quite a long time before the enzymes responsible for its synthesis were identified and cloned, and their mechanisms of activation elucidated.

Following the convincing demonstration that cGMP production takes place both in the soluble and particulate fractions of rat heart and lung homogenates [2,3], the enormous number of studies carried out since then has established that the cyclic nucleotide is produced by two receptor-enzymes that differ in cellular localization, molecular structure, and mode of activation.

Particulate guanylyl cyclases (pGC) represent a family of classical transmembrane receptor-enzymes that comprises 7 members (GC-A to GC-G) encoded by different genes and activated by different ligands, which are expressed in many tissues and cell types where they regulate numerous physiological processes [4,5,6]. Among these pGCs, GC-A, activated by atrial and brain natriuretic peptides (ANP and BNP, respectively), and GC-B, activated by the C-type natriuretic peptide (CNP), are expressed also in the brain.

All members of the pGC family function as transmembrane homodimers with each subunit basically composed by a N-terminal extracellular portion, a short α-helix hydrophobic transmembrane chain, a juxtamembrane domain, a Kinase Homology Domain (KHD), a hinge region, and a C-terminal catalytic domain. The extracellular N-terminal region represents the selective receptor, therefore showing the highest diversity among the seven pGC isoforms. While the endogenous ligands for GC-A to D have been identified (i.e., ANP, BNP, CNP, guanylin, and uroguanylin), no molecules have been found to bind the extracellular domain of GC-E to G. The intracellular KHD (also called ATP-Regulatory Module, ARM) [5] extends from the juxtamembrane domain to the catalytic region and shows a significant homology with several kinases, though it does not possess any phosphorylation activity. Indeed, the KHD/ARM needs to bind ATP and to be phosphorylated for pGC to properly produce cGMP in response to ligand binding, to regulate the switch of the receptor domain from a high- to a low-affinity state, and to mediate its desensitization process. The hinge region (also termed Signaling Helix Domain, SHD) [5] is necessary for both catalytic subunit dimerization and full cGMP-synthesizing activity, although not for all GC members [7]. Finally, the catalytic domain is the part of pGCs that converts GTP into cGMP. From a structural point of view, this region is highly conserved in both particulate and soluble GCs and shares homologies with the catalytic subunits of adenylyl cyclase. Moreover, as said above, two catalytic subunits are necessary for the cyclase activity. In addition to these mechanisms, it has been shown that the activity of GC-E and GC-F, which are expressed in the retina and involved in phototransduction, is regulated by calcium sensors guanylate cyclase activating proteins (GCAPs) in a calcium-dependent manner, being maximal at low calcium levels (1–10 nM) and getting suppressed at higher concentrations (200–500 nM) [5]. Additionally, GC-E activity can be boosted again when calcium exceeds 1 μM due to the action of S100B, another stimulatory calcium sensor [5,7]. Last but not least, activity of some GC members (i.e., GC-D, GC-E, GC-G) was found to be stimulated by bicarbonate ions [4,5,7].

Soluble guanylyl cyclase (sGC) is a cytosolic enzyme that has definitely changed the classic concept of cell-to-cell signaling, being activated by nitric oxide (NO), the first gaseous molecule identified as an intra- and inter-cellular transmitter in mammals. sGC is a heterodimer formed by the assembly of a short (α) and a long (β) subunit, both necessary for the enzymatic activity [6,8,9]. Although two isoforms have been isolated for each subunit (α_1–2_, β_1–2_), the vast majority of sGCs expressed in mammals shows the α_1_β_1_ composition that confers the highest specific activity in comparison to other possible arrangements. sGC is localized in almost all tissues with the highest relative expression in various regions of the central nervous system (CNS) [10].

As for the structure, each subunit comprises four domains that, according to current nomenclature, are termed Heme-Nitric Oxide Oxygen binding (H-NOX) domain, Per-Arnt-Sim (PAS) domain, Coiled-Coil (CC) domain, and a Catalytic (CAT) domain. The H-NOX domain is present in both α and β subunits but only the latter has the capacity to bind a Fe^2+^-containing heme group through a histidine residue. This part of the β subunit represents the receptor for NO that binds to the histidine-bound pentacoordinate iron to form a transient hexacoordinate histidine-iron-nitrosyl complex that, in turn, quickly stabilizes into a pentacoordinate iron-nitrosyl heme group by cleavage of the iron-histidine bond, a conformational change that is thought to be instrumental to initiate the signal for the production of cGMP. The exact functions of the PAS and CC domains are not fully elucidated but they have been implicated in the heme insertion into the β subunit, in the dimerization process, in the modulation of NO affinity and in the signal transmission from the H-NOX to the CAT domain. The C-terminal CAT domains of the α and β subunits form the catalytic site of the heterodimer sGC enzyme, with the active pocket localized at the interface of the two subunits, which are both responsible for GTP binding. Although many studies have been carried out to establish how the CAT domain activity is modulated by NO, the exact mechanism has not been unveiled so far. Yet, the large body of evidence accumulated to date clearly indicates that the stimulation of sGC by NO is not a simple on-off process but rather the consequence of complex interactions among the different domains that result in subtle conformational changes maximizing cGMP production [9,11].

Once synthesized by either pGC or sGC, cGMP then translates extracellular signals to target cells by networking with three main functional partners: protein kinase G (PKG), cyclic nucleotide gated channels (CNGCs), and cGMP-regulated phosphodiesterases (PDEs).

PKG is a serine/threonine kinase that exists in two families PKG I and PKG II, the former showing the two splice variants PKG Iα −Iβ [12]. Although PKG and the cAMP(cyclic adenosine-3′,5′-monophosphate)-activated kinase (PKA) show a strong structural homology, at variance with PKA, the regulatory and catalytic domains of PKG are joined together in a single protein that needs to homodimerize to express a fully functioning activity. Both PKG families show a similar basic structure with a regulatory domain sited at the N-terminal region and a catalytic domain sited at the C-terminus. The regulatory domain contains an extended leucine zipper motif (having a dimerization and localization function) that is followed by an autoinhibitory/autophosphorylation region that modulates the enzymatic activity. After this subdomain, there are two cyclic nucleotide-binding sites (CNB-A and CNB-B) arranged in tandem that are necessary for the activation of the kinases and show different affinity for cGMP. Finally, the catalytic domain comprises two main subregions: the first one binds ATP and modulates the sensitivity of PKG activation to cGMP concentrations, whereas the second one represents the protein substrate binding site. With regard to activation mechanisms, it is generally accepted that, in the inactive state, the catalytic domain at the C-terminal region of each monomer interacts with the respective autoinhibitory sequence of the regulatory domain to form a bended structure that prevents the binding of substrate proteins. When cGMP binds to the CNBs, the interaction with the autoinhibitory region is relieved and the catalytic domain is moved away leading to the opening of the bended structure, thus allowing association to and phosphorylation of target proteins.

CNGCs belong to the superfamily of voltage-gated ion channels, though from a functional point of view they are pure ligand-gated channels as they are activated only by the binding of cAMP or cGMP [13,14]. Differently from the majority of this type of channels, however, the ligand-binding site of CNGCs is located in the intracellular side of the plasma membrane. After their discovery in rod photoreceptors, a large number of studies have investigated their structure and functions, as well as their localization, showing that they are particularly abundant within the CNS. CNGCs are heterotetrameric complexes composed by the assembly of A and B subunits that present multiple splice variants and form non-selective channels allowing the influx of Na^+^, K^+^, Ca^2+^, and Mg^2+^ upon activation by cAMP or cGMP. To date, four different A (CNGA1-4) and two B (CNGB1, 3) subunits have been identified which show the same main topology, with the N- and C-termini localized in the intracellular side of the plasma membrane, six transmembrane domains (S1–6), a reentrant pore loop between S5 and S6, and a C-linker connecting S6 to the intracellular cyclic nucleotide binding domain (CNBD) placed just before the C-terminus. Their functions have been mainly related to olfaction and vision, since CNG channels are abundantly localized on olfactory neurons and in rod and cone photoreceptors. However, a wealth of studies has accumulated showing that these channels are widely distributed in different regions of the CNS, from spinal cord to cerebellum, hippocampus, hypothalamus, and cortex, and are present both on neurons and astrocytes where they are responsible for modulating cell excitability [13].

**Figure 1:**
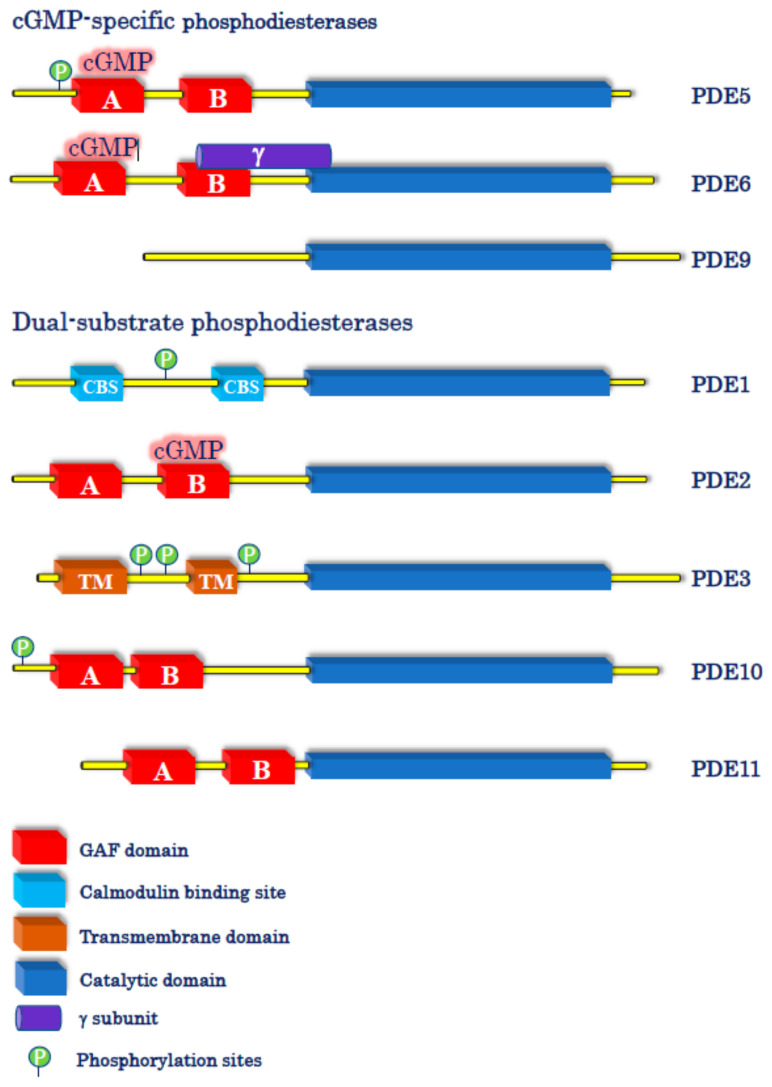
Schematic representation of cyclic guanosine-3′,5′-monophosphate (cGMP)-specific and dual-substrate phosphodiesterases (PDEs). The figure shows the three PDEs that specifically hydrolyze cGMP (PDE5, 6, and 9) and the five PDEs (PDE1, 2, 3, 10, and 11) that hydrolyze both cAMP and cGMP, although to a different extent.

The effects of cGMP are then terminated by its hydrolysis to GMP by specific PDEs. The superfamily of PDEs comprises 11 different genes, termed *PDE1* to *PDE11*, most of which express several splice variants (e.g., *PDE1A-C*; *PDE3A, B*; *PDE4A-D*), giving an overall number of approximately 100 enzyme isoforms [15]. Among all these family members, 3 are specific for cAMP (PDE4, 7 and 8), 3 are specific for cGMP (PDE5, 6, and 9) while PDE1, 2, 3, 10, and 11 metabolize both cyclic nucleotides, although with different preference ratios (Figure 1).

PDE5A and PDE6 (A, B, and C) show a similar basic structure, as both enzyme families possess a catalytic domain selective for cGMP hydrolysis in the C-terminal region and amino-terminal GAFA/B domains, with the former having high affinity in binding the cyclic nucleotide to stimulate the enzymatic activity. In the case of PDE5A, however, cGMP binding to GAF A is also regulated by PKG-mediated phosphorylation of a serine residue in the N-terminal region that further increases the affinity for cGMP. At variance with PDE5A, PDE6 activity is regulated by an inhibitory γ-subunit, and the enzyme is catalytically active as a homo- or heterodimer comprising α and β subunits.

PDE9A represents the enzyme with the highest affinity for cGMP among all PDEs and shows a rather simple structure if compared to PDE5 and PDE6, as it has no regulatory GAF domains, PKG phosphorylation sites, nor γ-subunits.

As for their cellular localization, PDE5A and PDE6 (A, B, and C) are generally considered cytosolic enzymes, whereas PDE9A isozymes can be localized either in the cytosol (e.g., PDE9A5) or at the nuclear level (e.g., PDE9A1, which has a pat7 nuclear motif). In the CNS, PDE5A and PDE9A are widely distributed in different brain regions, especially in cerebral cortex, hippocampus, cerebellum, and basal ganglia, whereas PDE6 isoforms are almost exclusively confined to the retina (PDE6A and B in rods and PDE6C in cones) and pineal gland.

Besides regulating the activity of PDE5A in a feed-forward fashion, cGMP is also able to modulate other PDEs [15]. In particular, cGMP can, respectively, stimulate and inhibit the activity of the dual-substrate (cAMP/cGMP) PDE2 and PDE3 enzymes. In the first case, potentiation of PDE2 activity is due to the allosteric effect of cGMP when it binds the GAF-B domain of the enzyme, thus leading to the enhancement of cAMP hydrolysis. For PDE3, the inhibition of cAMP degradation by cGMP occurs on the basis of substrate mutual competition for the enzyme when cGMP levels significantly increase [15].

Importantly, there is increasing evidence that, like for the cAMP-mediated signaling, also the different members of the cGMP pathway (particularly PDE5 and PDE9) are organized in precise signalosomes within discrete cellular microdomains to subserve different physiological functions [16].

## 2. The cGMP System in Memory Processes

Among the extraordinary brain processes that drive our everyday life, memory is certainly one of the most amazing and fascinating, yet extremely complex.

Following its first description by Bliss and Lømo [17], long-term potentiation (LTP) is today unanimously considered the form of synaptic plasticity that plays a critical role in memory formation and consolidation. Although this phenomenon of long-lasting increase of synaptic strength can be observed in different brain regions, hippocampal LTP has been the most thoroughly investigated and dissected out in a myriad of cellular and molecular determinants. Among these, the cGMP system has been consistently shown to be instrumental for the induction and expression of hippocampal LTP, as well as for memory formation and consolidation [18].

Indeed, since the early ’90s several studies have shown that blockade of NO formation by pharmacological treatments or genetic manipulations results in the inhibition of hippocampal LTP, although with different sensitivity depending on the type of stimulation used. Interestingly, studies with NO synthase (NOS) inhibitors and knock-out mice have revealed that both neuronal and endothelial isoforms of NOS (nNOS and eNOS, respectively) seem to be involved in hippocampal LTP. Furthermore, several studies have reported that pharmacological inhibition or genetic deletion of NOS is accompanied with a significant impairment of learning and memory, as assayed in behavioral tasks, such as radial arm maze, Morris water maze, passive avoidance, and elevate plus maze.

Similarly, blockade of sGC activity by the selective inhibitor ODQ was reported to dampen hippocampal LTP [19] and to alter memory functions [20]. On the contrary, the sGC activator YC-1 has been shown to enhance LTP [21] and ameliorate memory in adult and aged rodents [22].

With regards to PKGs, a first study in the early ‘90s proposed their role in hippocampal LTP [23] that was subsequently supported by other investigators showing that PKG inhibitors were able to reduce this process of synaptic plasticity [24]. As for the role of the two kinase isoforms, PKGI seems to be mainly involved in the presynaptic mechanisms of LTP [24], whereas PKGII has been found to act at the postsynaptic level [25]. However, mice with a selective deletion of PKGI in the hippocampus show no alterations of spatial reference and contextual memory, despite an impaired LTP under repeated stimulations [26]. On the contrary, PKGII knockout mice show a significant deficit in learning and memory functions that, however, could be ascribed to signaling alterations in the pre-frontal cortex rather than in the hippocampus [25,27].

The role of CNG channels in LTP and memory has been scarcely investigated. It has been reported that hippocampal LTP amplitude is markedly reduced in mice lacking the olfactory CNG channel 1 (OCNG1) [28]. At variance with those results, it was later observed that LTP is surprisingly enhanced in the hippocampus of CNGA3 null mutant mice, without significant changes in hippocampus-dependent learning and memory functions [29].

Finally, several studies have shown that also cGMP-specific PDEs are involved in the modulation of hippocampal LTP and memory formation in physiological conditions. For instance, it has been shown that hippocampal LTP is increased in adult mice treated with the PDE5 selective inhibitor sildenafil [30]. Moreover, bath application of sildenafil or vardenafil to adult mouse hippocampal slices has been reported to switch the relatively short-lasting early LTP (E-LTP) into the long-lasting late LTP (L-LTP) [31,32]. PDE5 inhibitors have also been shown to possess memory-enhancing properties in a variety of behavioral tests on healthy rodents and non-human primates [33]. Similarly, PDE9 inhibitors were found to facilitate hippocampal LTP and to ameliorate memory functions in adult and aged rodents [34,35].

## 3. The cGMP System in Alzheimer’s Disease

There are almost 50 million people suffering from dementia worldwide and Alzheimer’s disease (AD) certainly represents the most common cause, accounting for up to 70% of all forms of dementia. The currently available drugs for the symptomatic treatment of AD are three acetylcholinesterase inhibitors (donepezil, rivastignime, galantamine) and one NMDA receptor antagonist (memantine) that, however, have rather limited efficacy and do not modify the disease progression. Therefore, there is a compelling urge to find more effective treatments.

Multiple lines of pre-clinical and clinical evidence indicate that the cGMP system is markedly altered in AD. Actually, transgenic murine models of the human pathology, as well as wild-type animals injected with β-amyloid (Aβ) or tau, show significant impairments in hippocampal LTP and hippocampus-dependent memory [36]. Among the multiple potential cellular and molecular mechanisms, downregulation of the NO/cGMP/PKG pathway has been proposed to play a key role in the synaptotoxic effects of Aβ and tau leading to memory impairment. Indeed, in vitro and in vivo pharmacological manipulations aimed at boosting cGMP levels, such as NO mimetic drugs [37,38], sGC stimulators [38], and PDE inhibitors [39,40], resulted in the rescue of Aβ/tau-induced synaptic and cognitive deficits. In addition, increasing cGMP levels with PDE5 inhibitors also leads to the reduction of Aβ load in transgenic models of AD, thus suggesting a potential neuroprotective effect [32]. However, it is worth noting that, under physiological conditions, the increase of cGMP by PDE5 selective inhibitors (e.g., sildenafil, vardenafil) leads to the enhancement of Aβ production that is necessary for the switch from E-LTP to L-LTP and for the beneficial effects on memory [32]. In addition, activation of the cGMP signaling cascade has also vasodilation and antiapoptotic/prosurvival effects and is able to promote adult neurogenesis, all factors that may well contribute to the positive outcomes on memory.

In AD patients, low levels of cGMP in the cerebrospinal fluid were found to correlate with the severity of memory impairment [41,42], and cerebral nNOS and eNOS immunohistochemical analysis revealed alterations in both enzymes. In particular, the number of nNOS mRNA-labeled neurons was significantly lower in AD hippocampus than in controls [43]. Similarly, a substantial loss of nNOS immunoreactive neurons was observed in the hippocampus of AD patients, whereas the opposite was found in hippocampal astrocytes, especially those surrounding Aβ plaques [44]. However, others reported an increase of nNOS mRNA reactive hippocampal neurons in AD [45]. In addition, elevation of eNOS was observed in AD cortical astrocytes that were associated with Aβ plaques [46]. More recently, a marked reduction of hippocampal nNOS and eNOS protein expression, as well as total NOS activity, was confirmed in AD patients in comparison with age-matched controls [47].

In line with the hypothesis of a correlation between NOS and AD, a 50% reduction in the activity of sGC has been reported in soluble fractions from the temporal cortex of AD patients [48], while low expression levels of sGC were observed in reactive astrocytes surrounding Aβ plaques [49].

Finally, in the temporal and entorhinal cortices of AD patients, the cGMP-specific PDE5 resulted increased at both mRNA and protein levels [41,50,51], whereas studies on PDE9 gave opposing results [41,52].

Interestingly, it has been recently shown that chemically-induced LTP was impaired in functional synapses isolated from parietal cortex samples of AD patients, an effect that was rescued by the PDE5 inhibitor vardenafil or the PDE9 inhibitor BAY-736691 when coupled to sGC activation [53].

Other dual-substrate PDEs, such as PDE2A and PDE10A, did not show any difference in AD brain [41,52].

## 4. Clinical Studies on cGMP-Enhancers

Although to date most of the research has focused the attention on PDE inhibitors [54], the cGMP system can be activated by different pharmacological approaches in order to evaluate its efficacy in enhancing cognition. Principal clinical studies testing the effects of cGMP enhancers on cognition are reported below and summarized in Table 1.

### 4.1. sGC Stimulators

Riociguat, a direct activator of sGC approved for the treatment of pulmonary hypertension, has been trialed on cognition in 20 healthy volunteers in a double-blind, placebo-controlled study using the cholinergic muscarinic antagonist biperiden (0.5 and 1 mg) to induce memory deficits [55]. Riociguat was not effective in any of the tests used to assess different types of memory (episodic, working, spatial), as well as in tasks evaluating attention and psychomotor activity. In addition, the sGC activator was not able to rescue the episodic memory impairment induced by biperiden. The inefficacy of riociguat could depend on the doses administered, perhaps too low to increase cGMP enough to stimulate memory processes.

In October 2020, a press release from Cyclerion Therapeutics announced interesting results from a Phase 1 translational pharmacology study on IW-6463, a novel sGC stimulator that is being developed for different cognitive disorders, including AD. The drug has been administered to elderly volunteers (≥65 years) once daily for 15 days in two different sessions separated by a washout period of 27 days [56]. The results showed that IW-6463 penetrates the blood-brain-barrier achieving desired CNS exposure and increasing cGMP cerebrospinal fluid levels, thus demonstrating target engagement. In addition, it revealed positive effects on several parameters associated with memory and attention processing.

### 4.2. cGMP-Specific PDE Inhibitors

Sildenafil, marketed in 1998 for the treatment of erectile dysfunction (ED), has been the first selective PDE5 inhibitor to be tested on central functions in clinical trials. In a first, double-blind study with a cross-over design [57], 10 healthy adult male subjects were administered with a single dose of 100 mg sildenafil and then tested for spatial auditory attention and visual word recognition associated with event-related brain potentials (ERP). Although no relevant effects were observed on behavior, sildenafil was able to induce changes in auditory ERPs indicative of increased focused attention and enhanced ability of target selection. In addition, sildenafil reduced the amplitude of early negative waveforms, a result claimed to suggest a possible effect on information processing. In another pilot study on 6 healthy male volunteers [58], sildenafil (100 mg) was administered as a single dose and a battery of 7 different psychophysical tests was used to assess central effects. No relevant effects of sildenafil were observed on most of the parameters analyzed, except for an improvement of the mean reaction time in the simple choice reaction test. Similarly, no cognitive improvement was observed in a double-blind, placebo-controlled study [59] where sildenafil (50 and 100 mg) was given to 15 schizophrenic patients who received a cognitive test battery immediately (1 h) and 48 h after drug administration.

More recently, the effect of a single oral dose of sildenafil (50 mg) has been revaluated on cerebral blood flow (CBF), cerebral metabolic rate of oxygen (CMRO_2_), and cerebrovascular reactivity (CVR) in AD patients [60]. The results showed that CBF and CMRO_2_ were significantly increased 1h after sildenafil administration, especially in medial temporal lobes, whereas CVR was decreased throughout the brain, thus indicating a clear improvement of cerebral perfusion and enhancement of oxygen consumption that could be beneficial for the CNS functions of AD patients.

In addition, a pilot study on a small number of AD patients [61] (5 males and 5 females) reported that a single administration of sildenafil (50 mg) is able to normalize the fractional amplitude of low frequency fluctuations (a measure of spontaneous neural activity) in the right hippocampus and in the left and right parahippocampal regions, although in the two latter cases the effect did not reach statistical significance. This normalization was not related to vascular effects and could help improve cognitive functions.

The PDE5 inhibitor vardenafil was tested on cognition in young healthy subjects in two different trials. A double-blind placebo-controlled study, with three-way cross-over design, enrolled 18 university students who received vardenafil (10 and 20 mg) and underwent auditory sensory gating test 85 min later [62]. Using the same experimental design, a second trial [63] evaluated memory and executive functions on 18 out of 40 students selected on the basis of their performance in a preliminary memory screening. In both studies, however, vardenafil failed to show any significant effect on memory, executive functions and information processing.

Another PDE5 inhibitor, with a selectivity profile similar to that of sildenafil and commercially available for ED, is named udenafil. In a preliminary trial [64], 27 patients diagnosed with ED and treated with udenafil (100 mg every 3 days for 2 months) underwent the Korean version of the mini mental state examination (K-MMSE) for general cognition, the Soul verbal learning test (SVLT) for episodic verbal memory and the Korean version of the frontal assessment battery (K-FAB) for frontal execution functions. Results showed that K-MMSE and K-FAB scores were significantly improved after udenafil treatment, whereas no differences were found in the SVLT total and delayed recall. In a subsequent double-blind, placebo-controlled study, 49 ED patients were randomized for an 8-week treatment with 50 mg udenafil and then examined for cognitive performance using the K-MMSE and the Seoul Neuropsychological Screening Battery [65]. Udenafil was able to significantly increase general cognition and attention/working memory and showed a trend of improvement in frontal execution functions.

Concerning PDE9 inhibitors, three studies failed to show significant effects on cognition in AD patients. A Phase 2, double-blind, placebo-controlled trial [66] was performed on mild to moderate AD patients who received the PDE9 inhibitor PF-04447943 (25 mg, twice daily) for 12 weeks. No significant differences were observed using the Alzheimer’s Disease Assessment Scale-cognitive subscale (ADAS-cog), the Neuropsychiatry Inventory, and the Clinical Global Impression (CGI)-Improvement scale. A few years later, a second PDE9 inhibitor (BI 409306) was tested on patients with prodromal or mild AD in two Phase II, double-blind, placebo-controlled trials, with a total of 427 patients completing the studies [67]. The drug (10, 25, 50 mg once daily or 25 mg twice daily) was administered for 12 weeks, but, also in this case, the analysis of the primary (changes in the Neuropsychological Test Battery *z*-score) and the secondary endpoints (changes in Clinical Dementia Rating scale-Sum of Boxes, ADAS-Cog11) did not show any beneficial effects of drug treatment.

### 4.3. Dual-Substrate PDE Inhibitors

PDE1 is able to hydrolyze cAMP and cGMP, with PDE1A/B having higher affinity for cGMP and PDE1C being equally active on both cyclic nucleotides [15]. Besides other mechanisms of action, vinpocetine is a selective PDE1 inhibitor with higher potency for PDE1A/B than for PDE1C [68].

In a trial with a double-blind, placebo-controlled, crossover design, 12 cognitively normal women were treated with vinpocetine (10, 20, or 40 mg, three times daily) for 2 days and then subjected to a battery of psychological tests that gave negative results, except for the Sternberg task for short-term memory, which showed a significant improvement in the 40 mg-treated subjects [69]. Nevertheless, in a successive open-label pilot study trialing different doses of vinpocetine (30, 45, and 60 mg/day) in 15 AD patients for 1 year, no improvement was observed in the CGI or in any of the psychometric tasks performed, and the cognitive decline rate was similar in treated and control patients [70]. Beneficial, though inconclusive, cognitive effects of vinpocetine (30 and 60 mg/day) were reported in three randomized, double-blind studies in subjects with dementia selected from the Cochrane Dementia & Cognitive Improvement Group’s Specialized Register [71]. Positive effects on cognitive decline were also reported in patients with stroke or mild cognitive impairment (MCI) and cerebral hypoperfusion following a 12-week or an 18-week treatment with vinpocetine [72,73]. Lastly, in a recent prospective analytical study [74], vinpocetine (5 mg, twice daily for 3 months) has been reported to ameliorate some cognitive domains in a Nigerian population of 56 subjects with cognitive impairment due to epilepsy or AD/vascular dementia.

PDE3 is another enzyme able to metabolize both cyclic nucleotides with superimposable affinity but at a very different rate, being the V_max_ for cAMP approximately 10-fold higher than that for cGMP [15]. Cilostazol, a selective PDE3 inhibitor approved for the treatment of intermittent claudication and peripheral arterial disease, has shown promising neuroprotective effects against Aβ -mediated toxicity and has been experimented in several clinical trials for cognitive-enhancing effects [75]. In a first open-label, pilot follow up study published in 2009 [76], it was reported that a 6-month add-on therapy with cilostazol (100 mg/day) improved the MMSE score in a small group of mild/moderate AD patients under donepezil treatment. In another pilot trial [77], the same dose of cilostazol improved the cerebral blood flow in 20 patients suffering from AD or cerebrovascular disease, and prevented the cognitive decline observed in the control group treated with aspirin or clopidogrel. At the same time, Taguchi and collaborators [78] published a retrospective analysis on 70 patients treated with cilostazol (mean dose was 130 mg/day, in the absence of any other anti-dementia therapy) and evaluated at least twice for MMSE with an inter-test period of more than 6 months. In all treated patients, cilostazol was able to prevent the decrease in MMSE observed in the control group. Moreover, a subgroup analysis showed that this effect occurred in patients with MCI but not in demented or cognitively normal subjects. A recent retrospective study [79] on 40 AD patients analyzed the effects of cilostazol both as monotherapy (mean dose 150 mg/day) and as add-on therapy with galantamine (mean dose 14 mg/day). The results indicated that, as monotherapy, cilostazol is able to ameliorate the score of MMSE and Revised Hasegawa’s dementia scale, and to potentiate galantamine-induced improvements when used as add-on therapy. The positive effects of cilostazol as an add-on therapy were confirmed in a case-control study on 30 AD patients under therapy with acetylcholinesterase inhibitors for at least 12 months [80]. Finally, cilostazol (50 mg, twice daily) has been tested on MCI patients in a 96-week double-blind, placebo-controlled trial to evaluate beneficial effects on MMSE and other cognitive secondary outcomes (COMCID) [81]. The clinical study has been completed, but the results have not yet been disclosed [82].

## 5. Conclusions

The enormous number of preclinical studies have undoubtedly demonstrated that the cGMP-signaling system is among the molecular determinants for learning and memory formation, as well as that it is an adequate pharmacological target to reverse synaptic plasticity impairment and memory deficits in in vitro and in vivo models of AD.

These clear-cut beneficial effects contrast with the inconclusive clinical data obtained with PDE5 inhibitors. However, it has to be noted that in most of the few trials present in the literature, these inhibitors (e.g., sildenafil and vardenafil) have been tested with an acute administration paradigm, at one or two doses (those effective on ED), in healthy subjects or in pathological conditions other than AD and, when AD, in patients with mild-to-moderate severity. The evidence that repeated administrations of udenafil gave significant effects on cognition clearly suggest that we need to analyze the potential cognitive-enhancing effects of PDE5 inhibitors following a chronic administration schedule. Like many pro-cognitive drugs, PDE inhibitors show hormetic-like dose-response relationships, being effective in a very limited range of doses [83]; therefore, more doses should be tested. In addition, more robust effects may be seen in patients with prodromal AD than in those in more advanced stages of the disease.

Regarding PDE9 inhibitors, this does not appear to be the case as the two studies with BI 409306 were conducted on patients with prodromal/mild AD chronically treated with 4 different doses. It could be argued that PDE9 is not an optimal target because it controls periplasmic levels of cGMP generated by the natriuretic peptide/pGC system, which seems to be upregulated in AD, but not the cytoplasmic cGMP fueled by the NO-stimulated sGC, which is involved in memory formation and is compromised in AD [40,84]. However, it cannot be ruled out that other doses or combination with other PDE inhibitors (see below) could result in beneficial effects on cognition.

In addition, the cAMP system is essential for the processes of memory formation/consolidation [85,86], and, indeed, the beneficial cognitive effects obtained by boosting the cGMP system seem to require a fully functional cAMP signaling [31] that, on the contrary, is down-regulated in AD [84]. Thus, a combined therapy aimed at inhibiting PDE5 and PDE4 enzymes (especially some isoforms, such as PDE4D) could be more effective in ameliorating cognitive deficits in AD patients, as already shown in aged rodents [87]. In fact, the more promising results obtained by inhibiting the dual-substrate PDE3 seem to point to this direction. Furthermore, since cGMP stimulates the activity of PDE2 that, in turn, can reduce cAMP levels, it is reasonable to assume that the cognitive-enhancing effects of a combined treatment with PDE4/5 inhibitors could benefit of an add-on therapy with PDE2 inhibitors.

However, the challenge of finding an effective drug therapy for memory deficits in AD is made increasingly difficult by the fact that we are just starting to understand how complex cyclic nucleotide signalosomes are, how they are differently compartmentalized in neuronal and non-neuronal cells, and how they can modulate different cognitive functions in a cell-specific manner.

## Figures and Tables

**Table 1:** cGMP enhancers in clinical studies on cognitive performance.

Drug	Mechanism	Main Outcomes	Refs
Riociguat	sGC activator	No effect	[55]
IW-6462	sGC activator	Positive effects	[56]
Sildenafil	PDE5 inhibitor	Not conclusive effects	[57,58,59,60,61]
Vardenafil	PDE5 inhibitor	No effect	[62,63]
Udenafil	PDE5 inhibitor	Positive effects	[64,65]
PF-04447943	PDE9 inhibitor	No effect	[66]
BI 409306	PDE9 inhibitor	No effect	[67]
Vinpocetine	PDE1 inhibitor	Possible positive effects	[68,69,70,71,72,73,74]
Cilostazol	PDE3 inhibitor	Positive effects	[75,76,77,78,79,80]

sGC: soluble guanylyl cyclase; PDE: phosphodiesterase.
